# Lattice-strained Na-ZnFe_2_O_4_ catalyst boosting CO_2_ hydrogenation to long-chain olefins

**DOI:** 10.1039/d6sc00447d

**Published:** 2026-04-20

**Authors:** Xinyan Ai, Chengchao Liu, Zhe Li, Yuhua Zhang, Sixu Liu, Haifeng Xiong, Jinlin Li

**Affiliations:** a Key Laboratory of Catalysis and Energy Materials Chemistry of Ministry of Education & Hubei Key Laboratory of Catalysis and Materials Science, South-Central Minzu University Wuhan 430074 China liuchchao@scuec.edu.cn lij@mail.scuec.edu.cn; b The State Key Laboratory of Physical Chemistry of Solid Surfaces, iChEM (Collaborative Innovation Center of Chemistry for Energy Materials), Department of Chemistry, College of Chemistry & Chemical Engineering, Xiamen University Xiamen 361005 China haifengxiong@xmu.edu.cn; c Innovation Laboratory for Sciences and Technologies of Energy Materials of Fujian Province (IKKEM) 4221 Xiangan South Road Xiamen 361102 P. R. China

## Abstract

Thermo-catalytic hydrogenation of CO_2_ to fuels and chemicals is an effective way to utilize CO_2_, but it faces significant challenges due to low CO_2_ conversion and product selectivity. Here, we report a lattice-strained FeZnNa catalyst synthesized *via* a mechanochemical method (FeZnNa-G), showing a high selectivity for C_4+_ long-chain olefins (C_4+_^=^) of 64.9% and C_2+_^=^ of 77.5% at a high CO_2_ conversion rate of 47.7%. We found that the lattice-contracted FeZnNa-G catalyst forms Na-enriched ZnO nano-islands on the surface after activation and a Na-ZnO/Fe_5_C_2_ structure with the presence of 97% Fe_5_C_2_, facilitating the formation of an HCOO* intermediate and enhancing CO_2_ activation. A high C_4+_^=^ space–time yield (STY) of 474.9 mg *g*_cat_^−1^·h^−1^ and an extremely low CO selectivity of ∼9.1% exhibited dual-high performance, significantly surpassing that in previous reports. This use of a lattice-strained catalyst offers a new strategy for the efficient conversion of CO_2_ into high-value olefins, and paves the way for potential industrial applications.

## Abstract 2

A lattice-strained Na-ZnFe_2_O_4_ catalyst evolves into a Na-ZnO/Fe_5_C_2_ structure after activation, enhancing CO_2_ activation *via* promoting HCOO* formation and achieving 47.7% conversion, 64.9% C_4+_^=^ selectivity, and an STY of 474.9 mg g_cat_^−1^ h^−1^.

## Graphical abstract

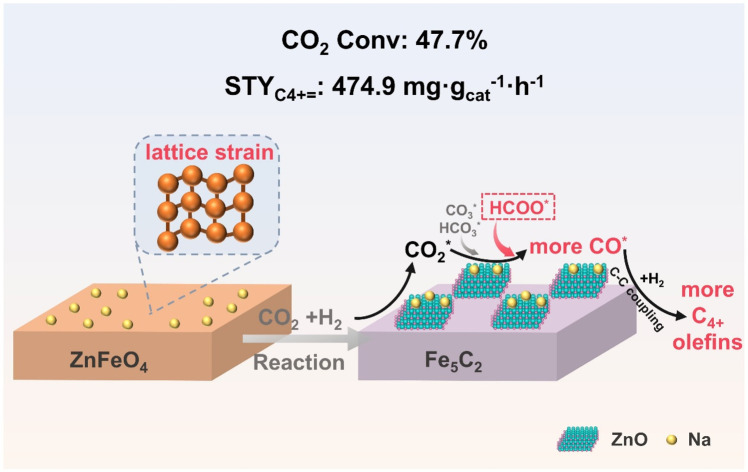

## Introduction

With the continuous growth of the global economy and energy demand, the massive consumption of carbon-based energy has led to a sharp increase in CO_2_ emissions, causing environmental issues such as global warming and ocean acidification.^1–3^ Driven by the pursuit of clean energy and carbon neutrality, the development of efficient and sustainable CO_2_ conversion technologies has become a research focus in the field of chemical engineering. Long-chain olefins serve as fundamental chemical feedstocks, with applications spanning critical sectors such as plastics, rubber, fine chemicals and fuel additives.^4–6^ Typically, C_4+_ olefins are obtained through fossil-based pathways, such as short-chain olefin polymerization, alkane dehydrogenation, and the recovery of petrochemical byproducts. In contrast, directly catalyzing the hydrogenation of CO_2_ into high-value long-chain olefins reduces atmospheric CO_2_ emissions and over-dependence on fossil fuels and provides an important pathway for carbon recycling.^7–12^

To date, oxide-zeolite bifunctional catalysts and Fe-based catalysts have demonstrated excellent performance in the hydrogenation of CO_2_ to hydrocarbons. Bifunctional catalyst systems exhibit high selectivity for olefins and aromatics, but are limited by low CO_2_ conversion and high CO selectivity (>40%).^13–16^ In contrast, Fe-based catalysts *via* the CO_2_-Fischer–Tropsch synthesis (CO_2_-FTS) pathway exploit the formation of iron oxides and iron carbides to activate the RWGS and FTS reactions, showing advantages in the production of long-chain hydrocarbons.^17–21^ However, the inherent stability of CO_2_ molecules makes activation challenging, and the thermodynamic equilibrium of the RWGS reaction limits the CO_2_ conversion efficiency. Moreover, the complex and variable phases of Fe catalysts during the reaction constrain the CO_2_-FTS pathway, presenting significant challenges in the efficient conversion of CO_2_ into long-chain olefins.

Fe_5_C_2_, as a critical active species commonly encountered in Fischer–Tropsch synthesis (FTS), exhibits excellent C–C coupling ability, facilitating the production of long-chain hydrocarbons.^22–25^ Studies have shown that introducing transition metals (*e.g.* Mn, Zn and Cu)^26–30^ and alkali metals (*e.g.* Na, K)^31–35^ as promoters into Fe-based catalysts can significantly enhance the formation of Fe_5_C_2_ and improve the catalytic performance. Notably, Zn and Na have received significant attention due to their pronounced effects. For example, Yang *et al.*^36^ reported that the formation of Fe_3_O_4_ and Fe_5_C_2_ as active phases led to high reaction activity and stability in CO_2_-FTS for the selective synthesis of olefins. Zn was reported to act as a structural promoter to improve the dispersion of Fe species, while Na serves as an electronic promoter to enhance CO activation, resulting in excellent olefin selectivity during CO hydrogenation.^37^ However, Zhang *et al.*^38^ suggested that ZnO activates CO_2_ to produce CO and Na, which primarily suppresses the secondary hydrogenation of olefins over an Na- and Zn-promoted iron catalyst. On a bimetallic Fe_5_C_2_-ZnO catalyst,^39^ the *in situ* formation of ZnO and highly dispersed FeO_*x*_ species on the catalyst surface enables the RWGS reaction to proceed, with the presence of FeC_*x*_ species promoting C–C coupling and alkene synthesis towards CO_2_ hydrogenation. Despite these reported studies on the FeZnNa system, the effects of structural evolution of Zn and Na promoters during reduction, activation, and reaction processes on the phase and performance of Fe catalysts remain unclear. Regulating the catalyst structure and elucidating the mechanisms of promoters and Fe species in Fe-based catalysts are key to achieving the efficient catalytic hydrogenation of CO_2_ to long-chain olefins.

In this study, we systematically compared spinel FeZnNa catalysts prepared by wet impregnation, co-precipitation, and mechanochemical methods. Through characterization techniques, such as high-resolution transmission electron microscopy, X-ray absorption spectroscopy, Mössbauer spectroscopy and density functional theory calculations, we determined the structural evolution of Na-ZnFe_2_O_4_ catalysts in CO_2_ hydrogenation, which favours the formation of long-chain olefins. The FeZnNa-G catalyst synthesized *via* a mechanochemical method exhibited significant lattice contraction strain, which facilitated reduction and carburization, achieving an Na-ZnO-enriched Fe_5_C_2_ catalyst with 96.7% Fe_5_C_2_ content.

## Results and discussion

### Catalytic CO_2_ hydrogenation performance

**Fig. 1:**
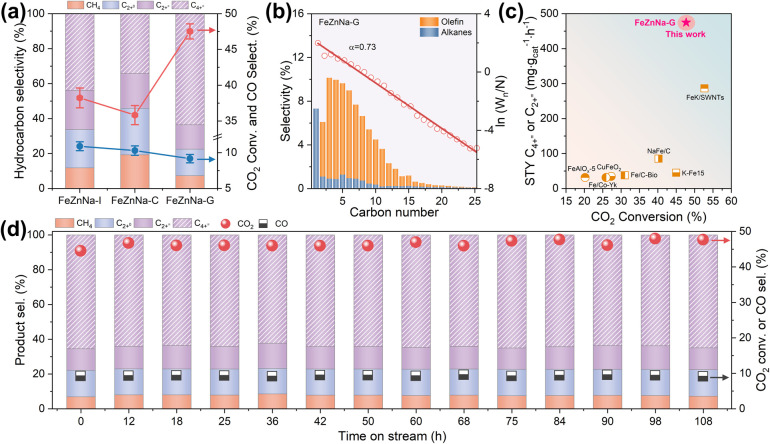
Catalytic properties. (a) Catalytic performance on Fe-based catalysts. (b) The product distribution and Anderson–Schulz–Flory (ASF) plots of FeZnNa-G catalysts. (c) The relationship of STY with CO_2_ conversion over various catalysts (see SI). (d) The stability of FeZnNa-G-catalyzed CO_2_ hydrogenation reaction at 340 °C, 2.0 MPa, 12000 mL g^−1^ h^−1^, H_2_/CO_2_ = 3.

FeZnNa-I, FeZnNa-C, and FeZnNa-G catalysts were prepared using impregnation, co-precipitation, and mechanochemical methods, respectively, with the elemental compositions listed in Table S1. The catalytic performance of the catalysts for CO_2_ hydrogenation was first evaluated at different reaction temperatures (Tables S2 and S3). As shown in the results, the FeZnNa-G catalyst exhibits higher catalytic activity than the other catalysts at the same reaction temperature. From the variation in product selectivity, relatively high CO selectivity is observed at lower temperatures. With increasing temperature, the CO selectivity gradually decreases, while the selectivity toward hydrocarbons correspondingly increases, indicating that the reaction over this series of catalysts mainly follows a modified RWGS-FTS reaction pathway. Under lower space velocity conditions, the CO_2_ conversion of the catalysts can reach more than 40%, whereas the CO selectivity decreases slightly. This is attributed to the longer residence time of reactant molecules on the catalyst surface, which facilitates the continuous occurrence of the subsequent FTS reaction. However, due to the thermodynamic equilibrium limitation of the CO_2_ hydrogenation reaction, increasing the reaction space velocity is more favorable for distinguishing the differences in catalytically active sites. Therefore, the catalytic performance for CO_2_ hydrogenation was systematically evaluated under reaction conditions of 340 °C, H_2_ : CO_2_ : N_2_ = 67.5 : 22.5 : 10, 2 MPa, and a GHSV of 12000 mL g^−1^ h^−1^. As shown in Fig. 1a and Table S3, the CO_2_ conversion over the FeZnNa-I and FeZnNa-C catalysts was 38.6% and 35.4%, respectively, with selectivities toward C_2+_^=^ olefins of 66.3% and 54.1%. Among these products, the selectivities for long-chain C_4+_^=^ olefins were 43.8% and 34.3%, respectively, indicating that the impregnation method is more favorable for the formation of long-chain olefins than co-precipitation. Notably, the mechanochemically obtained FeZnNa-G catalyst exhibited a markedly enhanced CO_2_ conversion of 47.7%. The selectivity to C_2+_^=^ products increased to 77.5%, within which long-chain C_4+_^=^ accounted for 83.7%, corresponding to a C_4+_^=^ selectivity of 64.9% (Fig. 1a and b). Meanwhile, the formation of undesirable CO and CH_4_ was effectively suppressed to 9.1% and 7.3%, respectively. No obvious deactivation was observed for any of the catalysts during 108 h of continuous stability testing (Fig. 1c and S1). Compared with previously reported catalysts under comparable reaction conditions (Fig. 1c and Table S3), the mechanochemically synthesized FeZnNa-G catalyst achieved a long-chain C_4+_^=^ space–time yield (STY) of 474.9 mg *g*_cat_^−1^·h^−1^, demonstrating a significant advantage. This FeZnNa-G catalyst not only achieved ‘dual-high’ performance in CO_2_ conversion and C_4+_^=^ product selectivity but also substantially suppressed the formation of low-value by-products such as CO and CH_4_.

### Structural characteristics and phase evolution of catalysts

**Fig. 2:**
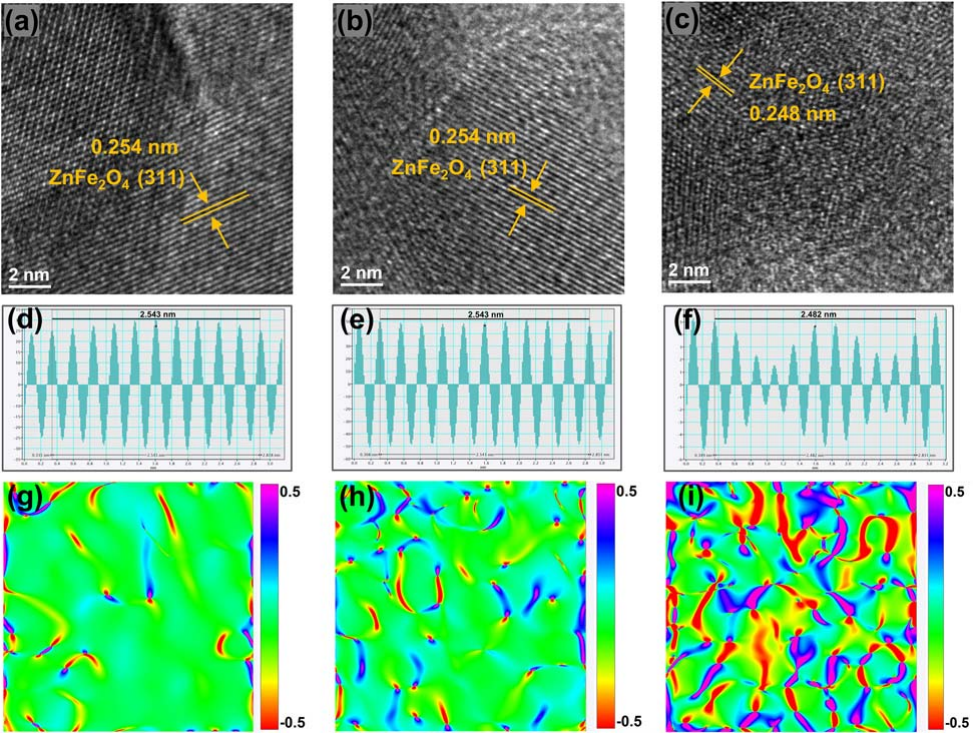
Structural characterization of the FeZnNa catalysts. HRTEM images of the as-prepared (a) FeZnNa-I, (b) FeZnNa-C and (c) FeZnNa-G catalysts. (d–f) Corresponding intensity profiles of a–c (g–i), corresponding strain maps of geometrical phase analysis (GPA) for HRTEM images.

X-ray diffraction (XRD) (Fig. S2) shows that the main phase of the catalysts is the spinel-type ZnFe_2_O_4_. The magnified patterns reveal that for the FeZnNa-G catalyst, compared to the other two FeZnNa catalysts, its characteristic diffraction peaks were shifted towards higher angles, indicating lattice contraction. Table S6 shows the physical properties of the catalyst, where the FeZnNa-G catalyst has a smaller particle size. The morphology of the FeZnNa catalysts was characterized by TEM. As shown in Fig. S3–S5, the catalysts were granular, and EDS analysis showed that the elements were uniformly dispersed. High-resolution transmission electron microscopy (HRTEM) images and interplanar spacing data (Fig. 2a–f) showed that the lattice spacing of FeZnNa-I and FeZnNa-C was 0.254 nm, while that of FeZnNa-G was 0.248 nm, corresponding to the (311) plane of ZnFe_2_O_4_. Notably, the lattice spacing in the FeZnNa-G catalyst was reduced, with distinct lattice dislocations and distortions. To better visualize the contracted state of the catalyst, strain maps were generated from the HRTEM images using geometric phase analysis (GPA, Fig. 2g–i), further confirming the lattice contraction strain in FeZnNa-G.

**Fig. 3:**
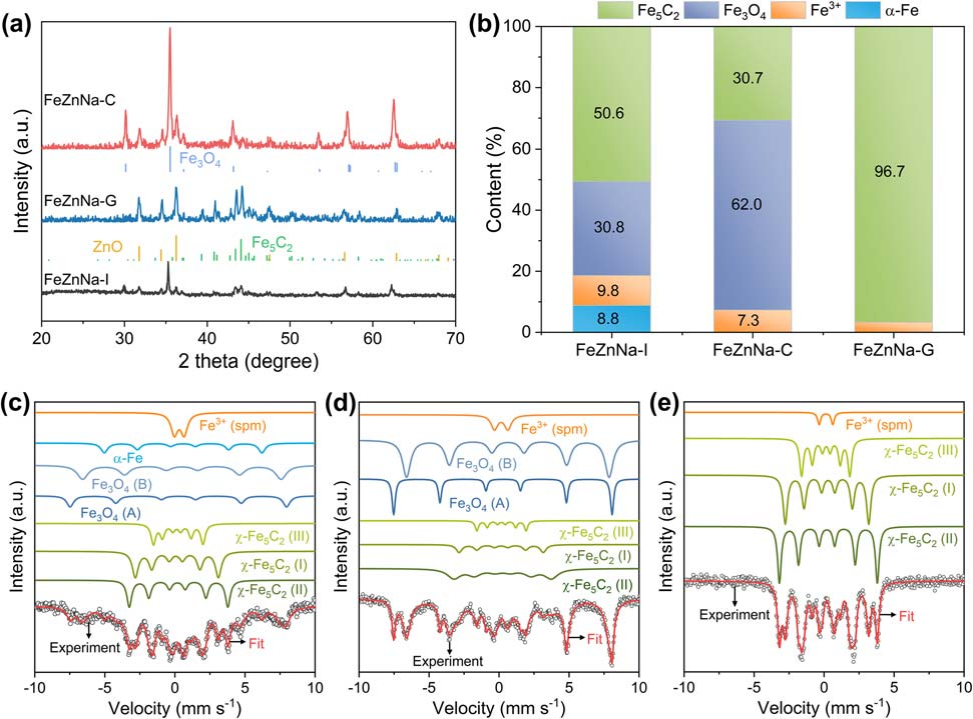
Composition and microstructure of catalysts after CO_2_ hydrogenation reaction. (a) XRD patterns of the spent catalysts. (b) Fe species content. Mössbauer spectra of the (c) FeZnNa-I, (d) FeZnNa-C, (e) FeZnNa-G catalysts after reaction.

Phase analysis of the catalysts after the reaction (Fig. 3a–e) shows that the catalysts underwent significantly different phase transitions during the reaction process. FeZnNa-I exhibits pronounced Fe_3_O_4_ characteristic peaks and faint Fe_5_C_2_ peaks, while FeZnNa-C displays prominent Fe_3_O_4_ peaks and weak ZnO peaks, and significant Fe_5_C_2_ and ZnO peaks are observed in FeZnNa-G. The Fe_5_C_2_ content in the catalysts after the reaction was 50.6%, 30.7%, and 96.7% for FeZnNa-I, FeZnNa-C, and FeZnNa-G, respectively (Table S7). It is generally believed that in the CO_2_-FTS process over Fe catalysts, Fe_*x*_O acts as the active center for the RWGS reaction, responsible for activating CO_2_ to produce CO, while Fe_*x*_C catalyzes the further hydrogenation of CO to hydrocarbon products.^41–44^ Fe_5_C_2_ with rich electronic properties has been confirmed as a highly active phase for converting CO into olefins.^27^ After the reaction, the FeZnNa-G catalyst with lattice contraction strain achieved a high Fe_5_C_2_ active phase content of 96.7%, facilitating C–C coupling and thus enhancing C_4+_^=^ olefin selectivity to 64.9% and achieving 47.7% CO_2_ conversion. The STY of C_4+_^=^ long-chain olefins reached 474.9 mg *g*_cat_^−1^·h^−1^, where the high content of Fe_5_C_2_ was key to the increased selectivity for long-chain olefins.

### Surface structural evolution during reaction

**Fig. 4:**
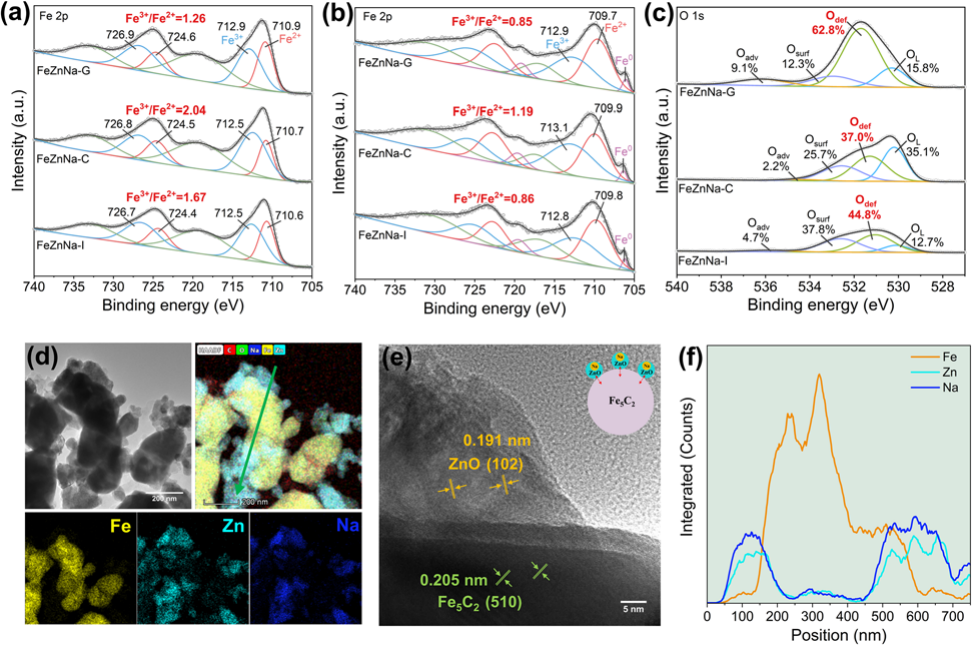
Fe 2p XPS spectra of the (a) as-prepared catalysts and (b) reduced catalysts. (c) O 1s XPS spectra of spent catalysts. (d) TEM images and corresponding elemental mapping. (e) HRTEM images and (f) line scanning profile of the arrow in (d).

The surface structural changes of the catalyst and their evolution during the reaction process were further examined. The X-ray absorption near-edge structure (XANES) results at the Fe *K*-edge (Fig. S6) show that the Fe chemical states in the catalysts are similar to those in Fe_2_O_3_. Comparison of the white-line peak intensities indicates that the oxidation state of Fe in FeZnNa-G is lower. The Fe 2p XPS results for fresh catalysts (Fig. 4a) show Fe^3+^/Fe^2+^ ratios of FeZnNa-C = 2.04 > FeZnNa-I = 1.67 > FeZnNa-G = 1.26, consistent with the XANES findings. Additionally, the lower Fe^3+^/Fe^2+^ ratio on the surface of the reduced FeZnNa-G suggests that its surface contains more low-valent Fe species, which is more conducive to the occurrence of the reduction and carbonization processes. Further analysis of the surface components of the catalyst before and after the reaction was conducted. The results in Tables S8 and S9 show that the Zn/Fe and Na/Fe ratios on the catalyst surface increased significantly after the reaction, indicating that Zn and Na migrated from the bulk phase to the surface during the reaction process. Notably, FeZnNa-G exhibits the highest migration ratios for Zn and Na. In addition, the O 1s spectrum in Fig. 4c shows that the peak area ratios of Odef in the FeZnNa-I, FeZnNa-C, and FeZnNa-G catalysts are 44.8%, 37.0% and 62.8%, respectively, indicating that the mechanochemical method facilitates the generation of more oxygen vacancies on the catalyst surface.

To reveal the structural changes on the surface of the catalyst, the morphology of the catalysts after the reaction was characterized (Fig. 4d–f). In the FeZnNa-G catalyst, uniformly dispersed ZnO particles form on the Fe_5_C_2_ surface, and EDS line-scan analysis performed across multiple regions shows that the intensities of Zn and Na are highly synchronized and are mainly enriched at the edge regions of the Fe particles (Fig.S9), indicating a strong interaction between Na and ZnO. As shown in the inset of Figure 4e, after the reaction, the closely associated Na-ZnO is well enriched on the Fe_5_C_2_ surface, forming the Na-ZnO/Fe_5_C_2_ structure. In contrast, for the FeZnNa-I catalyst, although Zn and Na are closely associated, ZnO is only partially enriched on the surface of the Fe species (Fig. S7). For the FeZnNa-C catalyst, ZnO is neither enriched on the Fe surface nor closely associated with Na, highlighting the significant differences in the structural evolution among the catalysts (Fig. S8).

### Reduction behavior and adsorption properties

The reduction behavior of the catalyst has a significant impact on the formation of the active phase. H_2_ temperature-programmed reduction (H_2_-TPR) results (Fig. S10) show that compared to FeZnNa-C, the FeZnNa-I catalyst exhibits a lower reduction temperature, while the FeZnNa-G catalyst demonstrates stronger reduction intensity, indicating that under a reduction temperature of 400 °C, the catalyst exhibits a higher degree of reduction, providing a larger amount of reducible iron oxide, thus supplying more metallic Fe precursors for the subsequent carburization process. After reduction at 400 °C with H_2_ for 2 h (Fig. S11), the phase of FeZnNa-I and FeZnNa-G shifted primarily from ZnFe_2_O_4_ to ZnO, FeO, and Fe, with FeZnNa-G showing a higher ZnO peak intensity, indicating that Zn is more easily segregated to form ZnO upon reduction in FeZnNa-G. Additionally, its relatively higher metal Fe characteristic peak further supported that the reduction degree was higher. In contrast, the phase of FeZnNa-C after reduction was mainly ZnO, Fe_3_O_4_, and Fe, retaining a significant amount of Fe_3_O_4_, suggesting that the reduction of Fe species in this catalyst is inhibited.

Clearly, the FeZnNa-G catalyst synthesized *via* the mechanochemical method facilitates the migration of Zn and Na to the catalyst surface, forming Na-ZnO nano-islands during reduction and activation. As electron-donating promoters, the surface enrichment of Zn and Na significantly influences the adsorption properties of the catalyst. To further investigate this observation, CO_2_ temperature-programmed desorption (CO_2_-TPD) tests were conducted on the post-reaction catalysts. As shown in Fig. S12a, FeZnNa-G exhibits significantly stronger desorption peaks, indicating that the enrichment of Na-ZnO on the Fe_5_C_2_ surface greatly enhances CO_2_ adsorption and activation. Moreover, the ability of the catalyst to adsorb CO is also critical in CO_2_ hydrogenation. Strong CO adsorption facilitates the second step of the FTS reaction, promoting further hydrogenation to long-chain hydrocarbons while reducing the formation of CO as a by-product.^45^ According to the CO temperature-programmed desorption (CO-TPD) results (Fig. S12b), FeZnNa-G presents strong desorption peaks, indicating higher CO adsorption capacity. This ability to adsorb more CO benefits the conversion of CO intermediates during the reaction, thereby improving C_4+_^=^ selectivity while simultaneously reducing selectivity for CO.

### Intrinsic role of lattice strain

**Fig. 5:**
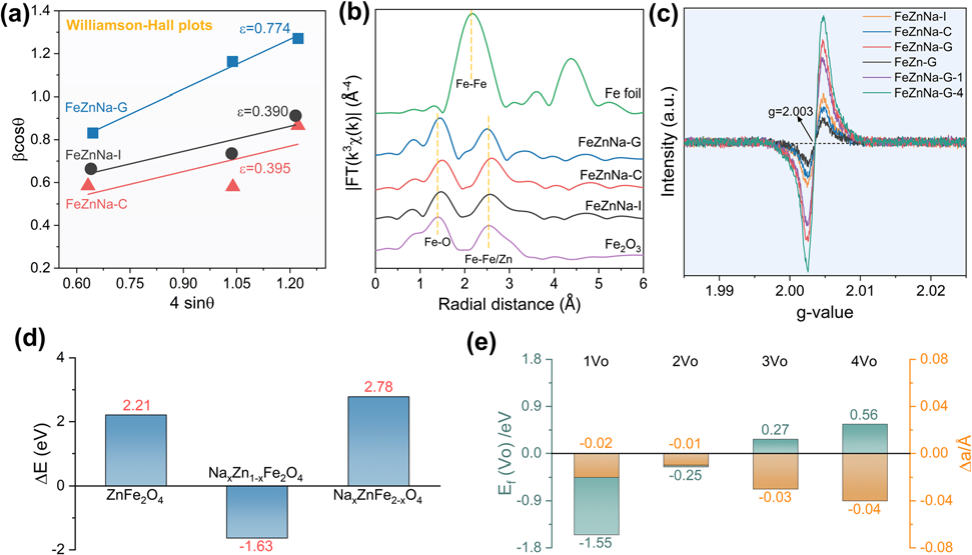
Structure characterization and DFT theoretical calculations. (a) Williamson–Hall plots of the Fe-based catalysts. (b) Fourier transformed EXAFS data for these FeZnNa catalysts. (c) Electron paramagnetic resonance diagram of the FeZn catalysts. (d) DFT calculation of the doping reaction energy (Δ*E*) of Na. (e) The formation energy (*E*_f_) of oxygen vacancy (V_O_) in Na-doping ZnFe_2_O_4_ and the lattice constant variation referring to the pristine ZnFe_2_O_4_.

From the above characterization results, it can be seen that the FeZnNa-G catalyst prepared by the mechanochemical method shows a higher content of Fe_5_C_2_. During the reaction, Zn and Na are more likely to migrate to the surface to form a stable Na-ZnO complex, and it has a higher degree of reduction and adsorption capacity for reaction molecules, significantly promoting the efficient generation of long-chain olefins from CO_2_ hydrogenation. More importantly, compared with other FeZnNa catalysts, the FeZnNa-G catalyst prepared by the mechanochemical method shows a certain degree of lattice contraction. The lattice strain values were calculated using the Williamson–Hall method.^40^ Here, the analysis is used mainly to provide a comparative evaluation of lattice strain among catalysts prepared by different methods. As shown in Fig. 5a, the FeZnNa-G catalyst exhibited a lattice strain (*ε*) of 0.774, significantly higher than that of FeZnNa-I (*ε* = 0.390) and FeZnNa-C (*ε* = 0.395), confirming significant lattice strain in the FeZnNa-G catalyst. Furthermore, the Fe *K*-edge extended X-ray absorption fine structure (EXAFS) results (Fig. 5b and Table S10) reveal that the Fe–Zn bond distance in FeZnNa-G (2.94 Å) is shorter than those in FeZnNa-I (2.96 Å) and FeZnNa-C (2.97 Å). This indicates that the lattice contraction strain induced by the mechanochemical method reduces the distance between Fe and Zn atoms in the catalyst. The closer Fe–Zn contact distance is expected to enhance the promoting effect of Zn on metallic Fe.

To clarify the fundamental cause of the strain generated by the catalyst, the mechanochemical method was employed to prepare FeZnNa-G catalysts with varying Na content, milling time, and milling method. The XRD patterns and strain data for the catalysts (Fig. S13 and S14) show that in the absence of Na, the strain value of the FeZn-G catalyst is 0.378, which is similar to those of the FeZnNa-I and FeZnNa-C catalysts prepared by impregnation and precipitation methods. However, when Na is introduced, the catalyst undergoes strain, and the strain increases with the increase in Na content. Meanwhile, the FeZn-Na-G catalyst was prepared by introducing Na later and using the mechanochemical method, and the strain value obtained was similar to that of the FeZnNa-G catalyst, indicating that the formation of strain is significantly related to Na.

Density functional theory calculations were performed to understand the lattice contraction induced by mechanical processing. The doping of sodium ions into the lattice of ZnFe_2_O_4_ spinel was considered by replacing the Zn/Fe atoms (Fig. 5d). The doping reaction energy (Δ*E*) of Na-to-Zn is negative (−1.63 eV), indicating the favorable replacement of the tetrahedral-coordinated Zn by an Na ion. However, it is positive (2.78 eV) for Na-to-Fe because of the higher binding energy (11.01 eV) of Fe with octahedral coordination than that (7.20 eV) of Zn. Moreover, the negative formation energy (*E*_f_ = −1.55 and −0.25 eV) of the 1st and 2nd oxygen vacancies (1*V*_O_ and 2*V*_O_) in Na-doped ZnFe_2_O_4_ spinel (Fig. 5e) indicates the favorable removal of oxygen by H_2_ reduction, leading to a slight contraction of the lattice constant (−0.02 to −0.01 Å). Characterization of oxygen vacancies further confirmed that the mechanical-chemical method facilitated the incorporation of sodium into the ZnFe_2_O_4_ lattice (Fig. 5f), inducing lattice contraction and generating oxygen vacancies. This enhanced the reduction degree of the catalyst, making the Fe oxide more prone to carbonization during the reaction to form Fe_5_C_2_, and facilitating the migration of Zn and Na to the surface during the reaction, ultimately forming a tightly bound Na-ZnO/Fe_5_C_2_ structure.

**Fig. 6:**
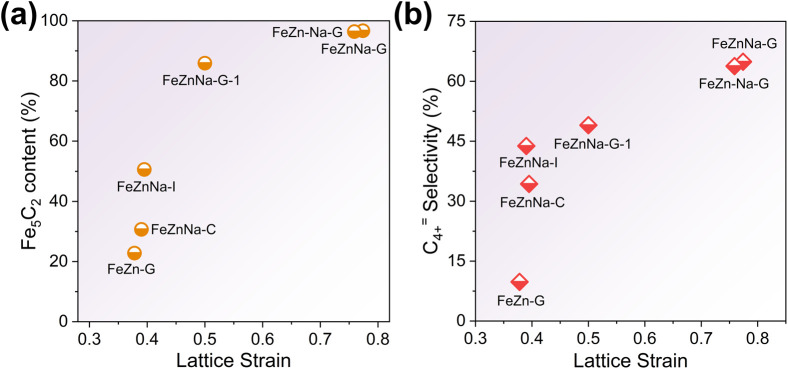
Correlation diagrams of lattice strain with (a) Fe_5_C_2_ content and (b) C_4+_^=^ selectivity.

To clarify the impact of lattice contraction strain on the formation of iron carbide and C_4+_^=^ selectivity, the CO_2_ hydrogenation performance of FeZnNa-G catalysts with varying contraction strains was tested (Table S11), and the Fe species content after reaction was characterized by Mössbauer spectroscopy (Fig. S15 and Table S7). The results show a positive correlation between Fe_5_C_2_ content and C_4+_^=^ selectivity with the contraction strain (Fig. 6a and b). When the strain (*ε*) reaches approximately 0.7, the Fe_5_C_2_ content stabilizes at more than 90%, and the C_4+_^=^ selectivity remains around 64%. These results indicate that the lattice contraction strain induced by the mechanical-chemical method facilitates the formation of Fe_5_C_2_. This is attributed to the fact that oxygen vacancies generated by lattice contraction enhance the degree of reduction, providing more metallic Fe precursors for the subsequent carburization process, thereby promoting the carburization of the catalyst during activation and increasing the Fe_5_C_2_ content. Given the superior chain growth capability of Fe_5_C_2_ as a vital active phase for long-chain olefin production, the lattice contraction significantly tailors the catalytic performance by modulating the carburization efficiency.

### Reaction mechanisms

**Fig. 7:**
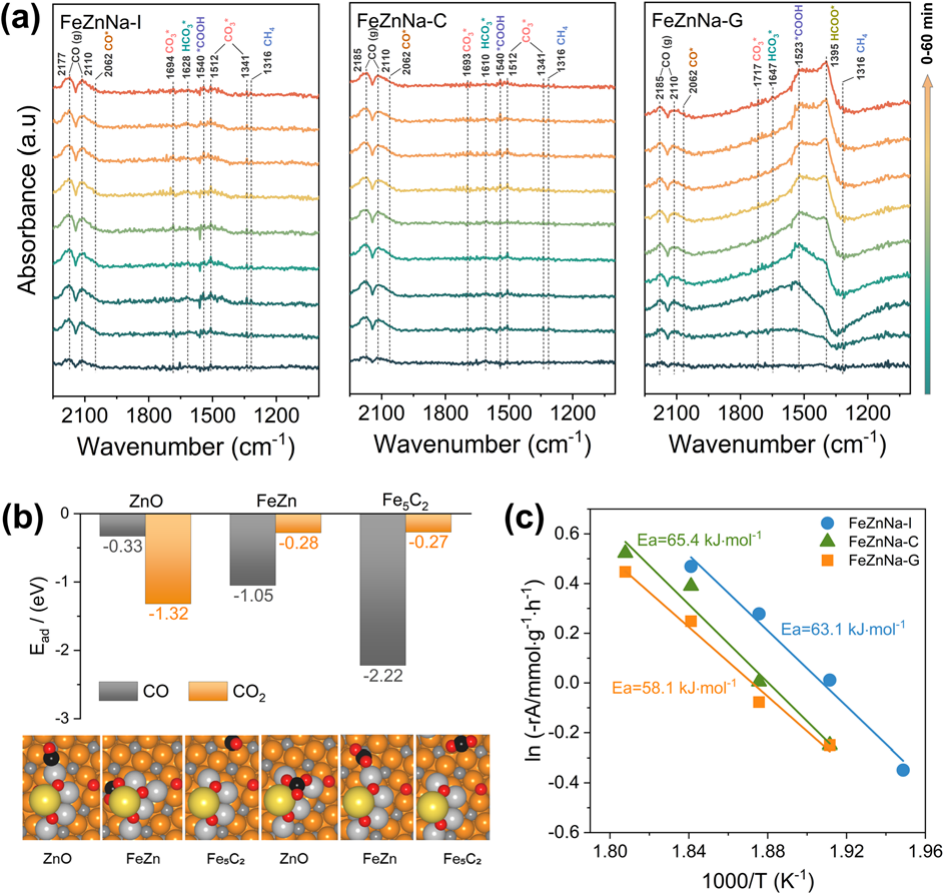
Reaction mechanism studies and schematic diagrams. (a) *In situ* DRIFTS spectra during CO_2_ hydrogenation of FeZnNa-I, FeZnNa-C and FeZnNa-G catalysts. (b) The CO/CO_2_ adsorption energy (*E*_ad_) and structures at different positions (ZnO, FeZn, Fe_5_C_2_) on the *χ*-Fe_5_C_2_ (510) surface. (c) Activation energies for CO_2_ conversion on catalysts.

To elucidate the reaction mechanism, an *in situ* DRIFTS experiment was employed to investigate the reaction pathways for the catalysts during the reaction (Fig. 7a), and the absorption bands at 2177 cm^−1^ and 2110 cm^−1^, and at 2062 cm^−1^, are attributed to gaseous CO and linearly adsorbed CO, respectively.^46^ Initially, there were virtually no adsorption peaks, but with increasing reaction time, the peak intensity continuously strengthened, indicating that the RWGS reaction generating the CO intermediate occurred first on the catalysts. The bands at 1341 cm^−1^, 1512 cm^−1^, and 1694 cm^−1^, and between 1610 and 1647 cm^−1^, are assigned to carbonate (
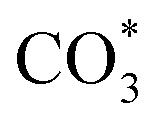
) and bicarbonate (
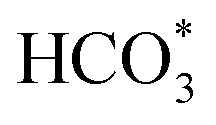
) intermediates, typically considered to be products of primary CO_2_ adsorption. The bands between 1523 and 1540 cm^−1^ correspond to carboxylate (COOH*), an important intermediate in the CO_2_ hydrogenation process.^47^ Notably, for the FeZnNa-G catalyst, a distinct adsorption band for formate (HCOO*) appeared at 1395 cm^−1.^^48–50^ Furthermore, the carboxylate (COOH*) peak at 1523 cm^−1^ was significantly enhanced initially but weakened over time as it was progressively converted into formate (HCOO*), suggesting that COOH* undergoes hydrogenation to form HCOO* during the reaction. Previous study^51^ has reported that the RWGS reaction of CO_2_ on ZnO proceeds mainly through the HCOO* intermediate. The intermediate reduces the apparent activation energy and enhances the catalytic activity. Clearly, the surface-enriched Na-ZnO plays a vital role in CO_2_ activation during the reaction. In the FeZnNa-G catalyst, as the reaction progressed, Na-enriched ZnO particles migrated to the Fe_5_C_2_ surface, forming the Na-ZnO/Fe_5_C_2_ structure. The surface-enriched Na-ZnO and Fe_5_C_2_ synergistically promoted the formation of HCOO* intermediates, providing a new pathway for the continuous activation of CO_2_. The activated CO was then adsorbed on Fe_5_C_2_ and underwent C–C coupling to produce long-chain olefins. DFT calculations further demonstrated the favorable adsorption of CO_2_ and CO on Na-ZnO-promoted Fe_5_C_2_ surfaces (Fig. 7b). CO_2_ prefers to adsorb on the ZnO site (*E*_ad_ = −1.32 eV), while CO favours binding to the Fe_5_C_2_ surface (*E*_ad_ = −2.22 eV). The highly dispersed Na-ZnO species on the surfaces of the FeZnNa-G catalyst favour sequential coupling between the CO_2_ and CO activation. In the CO_2_ hydrogenation reaction, CO_2_ activation is critical for sustaining the reaction, as more effective CO_2_ activation facilitates the subsequent reactions and increases hydrocarbon product selectivity. Kinetic analysis of the catalysts (Fig. 7c) revealed that the apparent activation energy of the FeZnNa-G catalyst synthesized by the mechanochemical method was 58.1 kJ mol^−1^, lower than those of FeZnNa-I (63.1 kJ mol^−1^) or FeZnNa-C (65.4 kJ mol^−1^). This finding further confirms that the synergy between surface-enriched Na-ZnO and Fe_5_C_2_ in the FeZnNa-G catalyst promotes the formation of HCOO* intermediate.

**Fig. 8:**
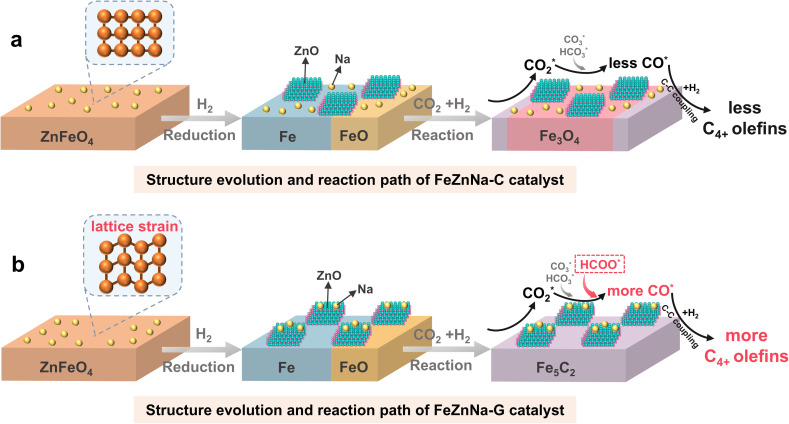
Schematic diagram of the evolution and reaction path of the catalysts.

Fig. 8 illustrates the relationship between catalyst performance and structural changes. The FeZnNa-G catalyst synthesized *via* the mechanochemical method undergoes lattice contraction induced by doping with Na under mechanical force, forming oxygen vacancies that enhance the degree of reduction and promote carburization. During activation, Zn and Na readily migrate to the surface, forming Na-ZnO complexes. The synergy between Na-ZnO and the high content of Fe_5_C_2_ facilitates the formation of HCOO* intermediate, providing a new pathway for sustained CO_2_ activation. The abundant Fe_5_C_2_ further efficiently promotes C–C coupling, enhancing selectivity for C_4+_^=^ long-chain olefins.

## Conclusions

To overcome the “seesawing” between activity and selectivity in CO_2_ hydrogenation to long-chain olefins (C_4+_^=^) over Fe-based catalysts, we synthesized a lattice-strained FeZnNa catalyst *via* a mechanochemical approach (FeZnNa-G). Compared to conventional FeZnNa catalysts prepared by wet impregnation and co-precipitation, the FeZnNa-G catalyst, synthesized under mechanical force, incorporates Na into the ZnFe_2_O_4_ spinel lattice, inducing lattice contraction and generating oxygen vacancies. These structural changes promote the reduction and carburization of the catalyst, leading to an extremely high content of Fe_5_C_2_ (96.7%) after the reaction. During the reaction, Zn and Na migrated to the surface, forming a closely bound Na-ZnO structure. The Na-ZnO/Fe_5_C_2_ catalyst generates a formate (HCOO*) intermediate, providing a new pathway for continuous CO_2_ activation and offering active carbon species for efficient C–C coupling on Fe_5_C_2_, enhancing the production of long-chain olefins. The FeZnNa-G catalyst achieved CO_2_ conversion of 47.7% and a C_4+_^=^ long-chain olefin selectivity of 64.9%, with a small amount of by-product CO of only 9.1%. The STY for C_4+_^=^ products reached 474.9 mg *g*_cat_^−1^·h^−1^, demonstrating significant advantages in converting CO_2_ to long-chain olefins. The lattice-strained catalyst prepared by mechanochemistry provides new insights and approaches for designing efficient heterogeneous catalysts.

## Author contributions

Xinyan Ai conducted all experiments and characterized the catalysts. Xinyan Ai, Chengchao Liu designed the experiments. Xinyan Ai, Chengchao Liu, Zhe Li, Haifeng Xiong wrote the manuscript. Chengchao Liu, Jinlin Li were responsible for funding application and supervised the project. Zhe Li, Yuhua Zhang, Sixu Liu contributed to the analysis and interpretation of the data.

## Conflicts of interest

There are no conflicts to declare.

## Supplementary Material

SC-OLF-D6SC00447D-s001

## Data Availability

The authors confirm that the data supporting the findings of this study are available within the article and its supplementary information (SI). Supplementary information is available. See DOI: https://doi.org/10.1039/d6sc00447d.
