# Supplementary material for: Lattice-strained Na-ZnFe2O4 catalyst boosting CO2 hydrogenation to long-chain olefins
Source: Chem Sci. 2026 Apr 20;17(22):11027–34. doi: 10.1039/d6sc00447d (PMC13108336; doi:10.1039/d6sc00447d)
Supplement: SC-017-D6SC00447D-s001 [file SC-017-D6SC00447D-s001.pdf]

Supplementary Information (SI) for Chemical Science.
This journal is © The Royal Society of Chemistry 2026

Supporting Information

Lattice strained Na-$ZnFe_2O_4$ catalyst boosting CO_2 hydrogenation to long-chain olefins

Xinyan Ai[†a], Chengchao Liu[†a,*], Zhe Li[†a], Yuhua Zhang[a], Sixu Liu[b,c], Haifeng Xiong[b,c,*], and Jinlin Li[a,*]

a. Key Laboratory of Catalysis and Energy Materials Chemistry of Ministry of Education & Hubei Key Laboratory of Catalysis and Materials Science, South-Central Minzu University, Wuhan 430074, China.
b. The State Key Laboratory of Physical Chemistry of Solid Surfaces, iChEM (Collaborative Innovation Center of Chemistry for Energy Materials), Department of Chemistry, College of Chemistry & Chemical Engineering, Xiamen University, Xiamen, 361005, China.
c. Innovation Laboratory for Sciences and Technologies of Energy Materials of Fujian Province (IKKEM), 4221 Xiangan South Road, Xiamen, 361102, P. R. China.

[†] These authors contributed equally to this work.

This PDF file includes:

Figures S1 to S16
Tables S1 to S11
SI References

Experimental Section

Catalysts preparation.

FeZnNa-I Catalyst. In a typical preparation, a solution of 0.3 mol/L $Fe(NO_3)_3·9H_2O$ and 0.1 mol/L $Zn(NO_3)_2·6H_2O$ was placed in a 500 mL beaker and subjected to an oil bath at 80 °C. NH_4OH was added dropwise with vigorous stirring until the pH reached approximately 9. The mixture was then allowed to age for 5 h, followed by washing with deionized water until neutral, and drying at 60 °C for 12 h. Subsequently, it was calcined at 350 °C for 4 h to obtain the FeZn powder sample. The FeZnNa catalyst, incorporated with alkali metal Na, was prepared using the initial wet impregnation method. Briefly, an appropriate amount of Na_2CO_3 aqueous solution was impregnated into the obtained FeZn powder, dried at 60 °C for 12 h, and then calcined at 350 °C for 4 h. The final catalyst was named FeZnNa-I.

FeZnNa-C Catalyst. Similarly, a solution of 0.3 mol/L $Fe(NO_3)_3·9H_2O$ and 0.1 mol/L $Zn(NO_3)_2·6H_2O$ was placed in a 500 mL beaker and subjected to an oil bath at 80 °C. NaOH solution was added dropwise with vigorous stirring until the pH reached approximately 9. The mixture was then allowed to age for 5 h, followed by washing with deionized water, and drying at 60 °C for 12 h. It was then calcined at 350 °C for 4 h. The resulting catalyst was named FeZnNa-C.

FeZnNa-G Catalyst. Based on the molar ratio of Fe:Zn:Na being 6:2:1, $Fe(NO_3)_3·9H_2O$, $Zn(NO_3)_2·6H_2O$, $NaHCO_3$, and NH_4HCO_3 were accurately weighed and mixed in a 250 mL Teflon-lined stainless steel balls milling tank, containing zirconium balls with a diameter of 1-3 cm. The ball milling process was carried out at 200 rpm for 2 h. The mixture was then dried at 120 °C for 12 h and calcined at 350 °C for 4 h. The resulting catalyst was named FeZnNa-G. Additionally, the $NaHCO_3$ content (adjusted proportionally), ball-milling time (30 min, 3 h), and preparation sequence (first synthesizing the FeZn precursor followed by ball milling Na into the precursor, this catalyst was named FeZn-Na-G) were varied to obtain additional FeZnNa-G catalysts with different lattice strains. The catalysts with 1%, 2% and 4% Na content was named FeZnNa-G-1, FeZnNa-G-2 and FeZnNa-G-4, respectively. The catalysts with 30 min and 3 h ball-milling time was named FeZnNa-G-30min and FeZnNa-G-3h.

Catalyst characterization.

The elemental content in the catalyst was analyzed using an Inductively Coupled Plasma Optical Emission Spectrometer (ICP-OES). The phase of the catalyst was examined using a Bruker-D8 X-ray powder diffractometer with Cu-Kα radiation (λ=0.154056 nm), operating at a voltage of 38 kV, and scanning the catalyst in the range of 10° to 80°. The Williamson-Hall approach was used to calculate the lattice strain value of the catalyst by the following eq. S1:

$$\beta\cos\theta = \frac{k\lambda}{D} + 4\varepsilon\sin\theta \quad \text{S1}$$

where β represent the half-peak width of the corresponding region measured by XRD. θ is the diffraction Angle of the corresponding peak position. The lattice strain ε of the catalysts can be calculated from the slope of the βcosθ vs. 4sinθ plots. The transmission electron microscopy (TEM), HAADF and element mapping images were obtained on a FEI Talos F200S system with an accelerationvoltage of 200 kV. The specific surface area (BET) and pore size distribution (BJH) of the catalyst were measured using a Micromeritics Tristar II 3030 N_2 physical adsorption-desorption instrument. The surface elemental distribution of the catalyst was analyzed using a VG Multilab 2000 X-ray Photoelectron Spectrometer (XPS) with an Al-Kα source, and an energy resolution of 0.47 eV (Ag-3d5/2), using the C1s peak binding energy of 284.6 eV for charge correction. Hydrogen Temperature-Programmed Reduction (H_2-TPR) was

conducted on a Zeton Altamira AMI-300 equipped with a thermal conductivity detector (TCD). Typically, the sample was purged with Ar at 50 °C for 30 min, followed by heating from 50 °C to 800 °C in a 5% H_2-Ar atmosphere, and held for 30 min. CO_2 Temperature-Programmed Desorption (CO_2-TPD) and CO Temperature-Programmed Desorption (CO-TPD) tests were also conducted on the same equipment. Briefly, after reduction with pure H_2 or 5% H_2-Ar at 400 °C for 2 h, the sample was purged with He for 30 min while cooling to 50 °C, then adsorbed with CO_2 or CO for 30 min, followed by purging with He for 30 min, and heating from 50 °C to 800 °C to test the adsorption of reactants and intermediates. It should be noted that, since only a TCD was employed, the desorption profiles reflect the overall desorption behavior (including CO_2, CO, and possibly H_2O formed during surface reactions) rather than exclusively CO_2 or CO. Therefore, the TPD data are mainly used for comparative analysis under identical testing conditions. Mössbauer spectra (MBS) of 57Fe were characterized using an SLD-500/SHI-850-05 Mössbauer spectrometer, with measurements taken in constant acceleration mode, using a ^{57}Co (Rh) radiation source, and velocity calibrated against α-Fe foil. Experimental data were automatically collected by computer and fitted with spectral lines using the least squares method. The X-ray Absorption Fine Structure (XAFS) of the fresh catalyst Fe-K edge was tested at the Hangzhou International Science and Innovation Center of Zhejiang University. The radiation was monochromatized using a Si double-crystal monochromator. XANES and EXAFS data were analyzed and processed using Athena and Artemis software. In situ Diffuse Reflectance Infrared Fourier Transform Spectroscopy (DRIFTS) spectra were acquired using a Thermo Scientific Nicolet iS50 FT-IR spectrometer. Before test, samples were in situ reduced at 400 °C under a pure H_2 atmosphere (10 mL/min) for 2 h, followed by a switch to Ar (10 mL/min) for a 30 min purge while cooling to 340 °C. Subsequently, a reaction gas mixture of 22.5% CO_2/66.7% H_2/10% Ar was introduced at a flow rate of 20 mL/min at a total pressure of 0.1 Mpa, and reacted at 340 °C, during which the infrared spectra were recorded throughout the reaction.

Computational details.

Density functional theory (DFT) calculations were performed using generalized gradient approximation Perdew-Burke-Ernzerhof (PBE) functional and projector-augmented wave (PAW) method as implemented in Vienna ab initio simulation package (VASP). Plane-wave kinetic energy cutoff of 520 eV was applied for the spin polarization calculations with the energy and force convergence criteria of 10-4 eV and 0.03 eV/Å, respectively. Gaussian smearing scheme (sigma = 0.05 eV) was used for modeling the semiconductor of bulk spinel with eight stoichiometric units of $ZnFe_2O_4$. Monkhorst-Pack k-point of 4 × 4 × 4 was applied for describing the cubic lattice of spinel (a = b = c = 8.45 Å). Grimme's dispersion (D3) correction was included for the DFT calculation with Hubbard U correction for Fe-3d state. The U value (5.3 eV) was determined to balance the accurate descriptions of bulk lattice (8.45 Å) of $ZnFe_2O_4$ and magnetic moment of Fe (4.2 μB) (Figure S14). The binding energy (Eb) of the Zn/Fe atom in spinel was calculated by removing a Zn/Fe atom from the bulk $ZnFe_2O_4$ forming a vacancy (V_{Zn}/V_{Fe})

$$E_b(Zn) = E(ZnFe_2O_4\text{-}V_{Zn}) + E(Zn) - E(ZnFe_2O_4)$$

$$E_b(Fe) = E(ZnFe_2O_4\text{-}V_{Fe}) + E(Fe) - E(ZnFe_2O_4)$$

in which the energy of isolated Zn/Fe atom was placed in a cubic box with a lattice of ca. 15 Å and calculated by gamma-only k-point. Sodium ion was doped into the bulk of $ZnFe_2O_4$ by replacing the Zn and Fe to obtain $Na_{x+y}Zn_{1-x}Fe_{2-y}O_4$.

$$ZnFe_2O_4 + (x+y)Na^+ \rightarrow Na_{x+y}Zn_{1-x}Fe_{2-y}O_4 + xZn + yFe$$

The doping reaction energy (ΔE) was calculated by

$$\Delta E = E(Na_{x+y}Zn_{1-x}Fe_{2-y}O_4) + xE(Zn) + yE(Fe) - E(ZnFe_2O_4) - (x+y)E(Na^+)$$

of which the negative value indicates the favorable Na doping. The formation energy (Ef) of oxygen vacancy (VO) was evaluated from the reduction with H2.

$$Na_{x+y}Zn_{1-x}Fe_{2-y}O_4 + zH_2 \rightarrow Na_{x+y}Zn_{1-x}Fe_{2-y}O_{4-z} + zH_2O$$

$$E_f = [E(Na_{x+y}Zn_{1-x}Fe_{2-y}O_{4-z}) + zE(H_2O) - E(Na_{x+y}Zn_{1-x}Fe_{2-y}O_4) - zE(H_2)]/z$$

of which the negative value indicates the favorable VO formation. The lattice constant variation (Δa) induced by Na doping and VO formation was evaluated by

$$\Delta a = a(Na_{x+y}Zn_{1-x}Fe_{2-y}O_4) - a(ZnFe_2O_4)$$

of which the negative/positive value indicates the lattice compression/expansion.

Furthermore, we select the reactive (510) surface of χ-Fe_5C_2 to investigate the favorable adsorption of CO/CO_2. The χ-Fe_5C_2(510) was modeled by a four-layered slab in (2 × 1) supercell with 80 Fe and 32 C in total, in which the top two layers were allowed to relax and the bottom ones were kept fixed. The vertical vacuum is ca. 15 Å thick. A zinc oxide cluster (Zn_4O_4) was deposited on the χ-Fe_5C_2(510) surface together with a Na nearby. Plane-wave kinetic energy cutoff of 400 eV and Monkhorst-Pack k-point of 3 × 3 × 1 were applied for the calculations with vertical dipole correction.

Catalytic performance test.

The CO_2 hydrogenation reaction performance of the catalyst was evaluated in a fixed-bed reactor. Initially, 0.1 g of the catalyst was loaded into a stainless-steel reaction tube. Prior to the reaction test, the catalyst was reduced under pure H_2 at 400 °C, 4000 mL·g^{-1}·h^{-1}, and atmospheric pressure for 2 h. After reduction, the temperature was lowered to 100 °C, and the gas was switched to a mixture of $CO_2/H_2/N_2$, with a molar ratio of the feed gas being 22.5/67.5/10, and the gas hourly space velocity (GHSV) was set at 12000 mL·g^{-1}·h^{-1}, reacting at 2 MPa and 340 °C. N_2 in the feed gas served as an internal standard gas for calculating the CO_2 conversion rate. All effluent gases were analyzed in real time using an Agilent Micro GC 3000 equipped with a TCD detector, with N_2 employed as an internal standard. Calibration curves for CO, CO_2, CH_4, and C_2-C_8 hydrocarbons were established using certified standard gases. A cold trap (-2-0 °C) and a hot trap (100 °C) were used to collect water, oil, and wax samples, respectively. Oil samples were analyzed on an Agilent 6890N GC-FID and quantified using standard solutions of n-alkanes, olefins, and alcohols. Water samples were analyzed on an Agilent 4890 GC-FID with alcohol standard solutions for calibration. Wax samples were first dissolved in carbon disulfide and then analyzed on an Agilent 7890A GC-FID, with response factors calibrated using long-chain alkane standards. The carbon numbers of all products were normalized for carbon balance calculations, which were above 96%, demonstrating the reliability of the tests. All performance measurements were repeated three times. The calculation methods for catalyst activity and product selectivity are as follows:

CO_2 conversion (X_{CO2}) was calculated by eq. S2:

$$X_{CO_2} = \frac{CO_{2in} - CO_{2out}}{CO_{2in}} \times 100\% \qquad \text{S2}$$

CO selectivity was calculated by eq. S3:

$$S_{CO} = \frac{CO_{out}}{CO_{2in} - CO_{2out}} \times 100\% \qquad \text{S3}$$

The selectivity of hydrocarbon was calculated by eq. S4:

$$S_{C_i} = \frac{C_i \times i}{\sum_{i=1}^{n} C_i \times i} \times 100\% \qquad \text{S4}$$

The space-time yield (STY) of $C_{4+}^{=}$ long-chain hydrocarbon products was calculated based on the molar number of CO_2 conversion products per gram of catalysts (g) per time (h).

$$STY\left(mg \cdot g_{cat}^{-1} \cdot h^{-1}\right) = \frac{X_{CO_2} \times S_{C_{4+=}} \times (1 - S_{co}) \times 12 \times 0.225 \times 14}{22.4} \times 1000$$

S5

Where CO_2in and CO_2out represent the molar numbers of CO_2 in the inlet and outlet reaction gases, respectively. CO_{out} represents the molar number of CO at the outlet. C_i represents the molar number of hydrocarbon products with a carbon number of i. $S_{C4+=}$ and S_{CO} is the selectivity of $C_{4+}^{=}$ and CO, respectively.

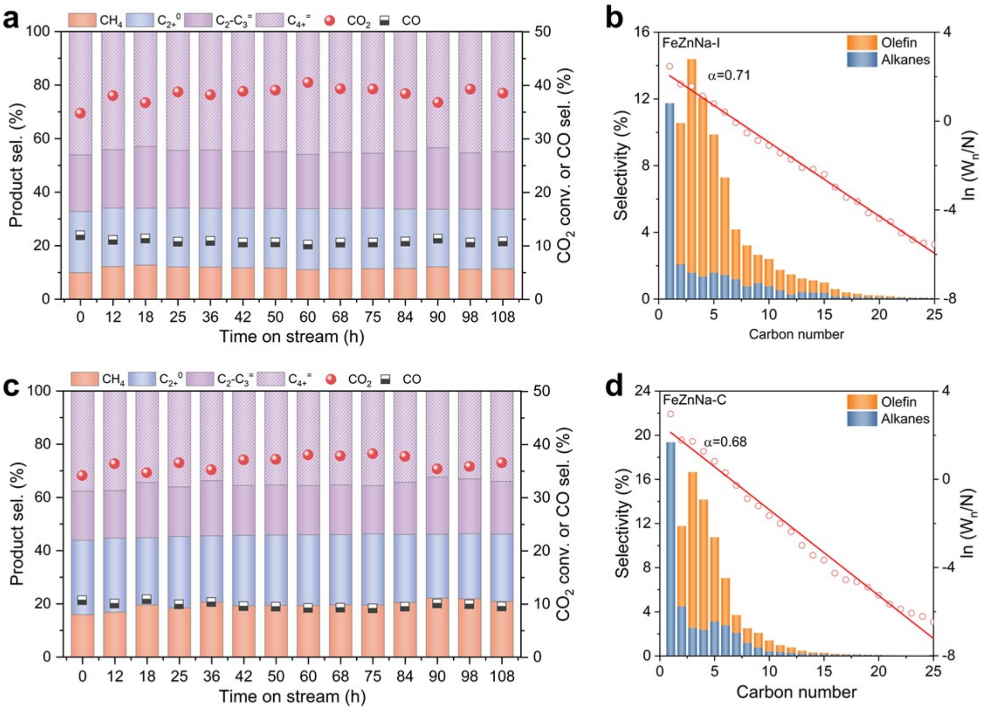

Fig. S1. The stability of catalysts CO_2 hydrogenation reaction and the product distribution and Anderson-Schulz-Flory (ASF) plots of (a-b) FeZnNa-I, (c-d) FeZnNa-C catalysts (340 °C, 2 MPa, H_2/CO_2 = 3:1, and 12000 mL· $g^{-1}\cdot h^{-1}$).

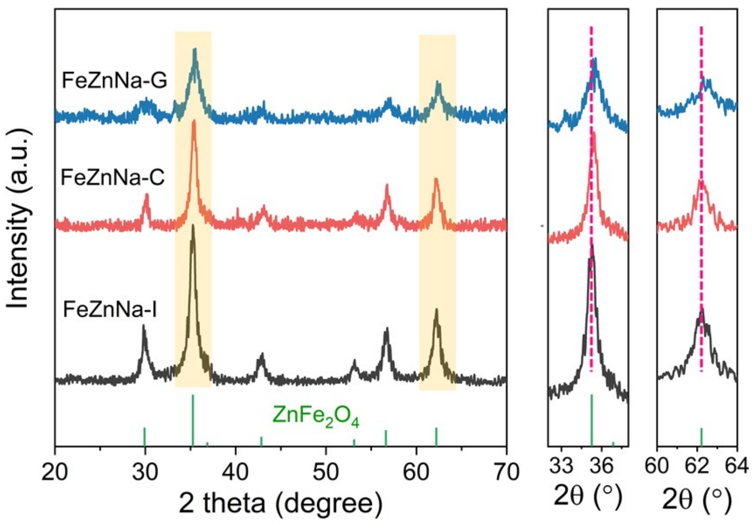

Fig. S2. XRD patterns of the as-prepared catalysts.

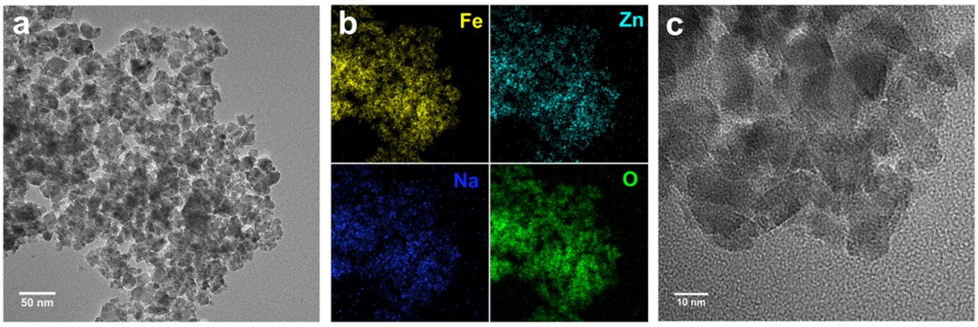

Fig. S3. TEM, corresponding EDS elemental maps and HRTEM images of the as-prepared FeZnNa-I catalysts.

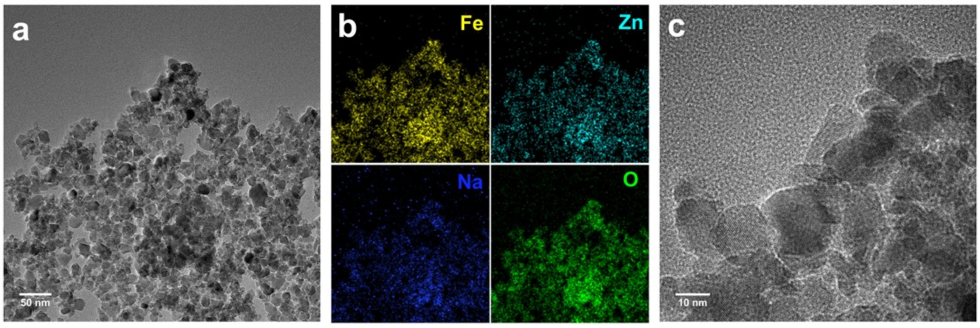

Fig. S4. TEM, corresponding EDS elemental maps and HRTEM images of the as-prepared FeZnNa-C catalysts.

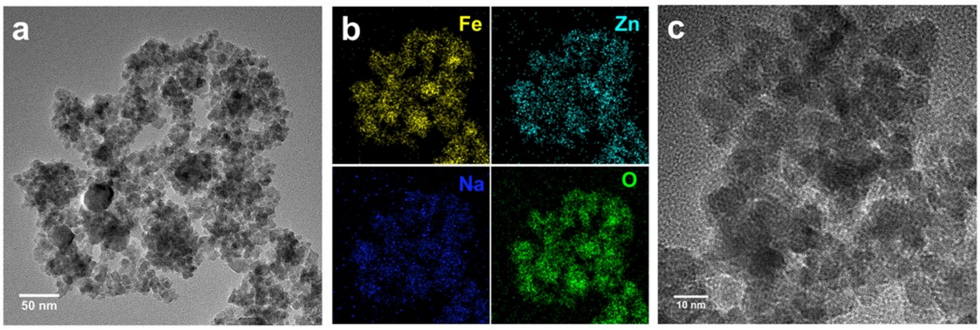

Fig. S5. TEM, corresponding EDS elemental maps and HRTEM images of the as-prepared FeZnNa-G catalysts.

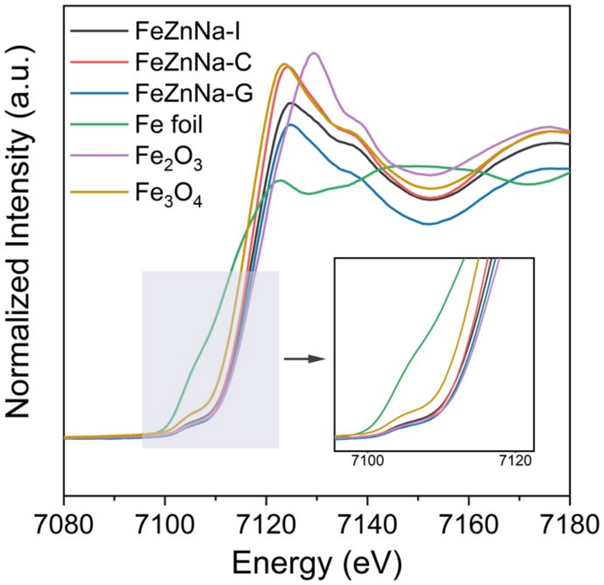

Fig. S6. XANES spectrum of Fe K-edge for the Fe-based catalysts.

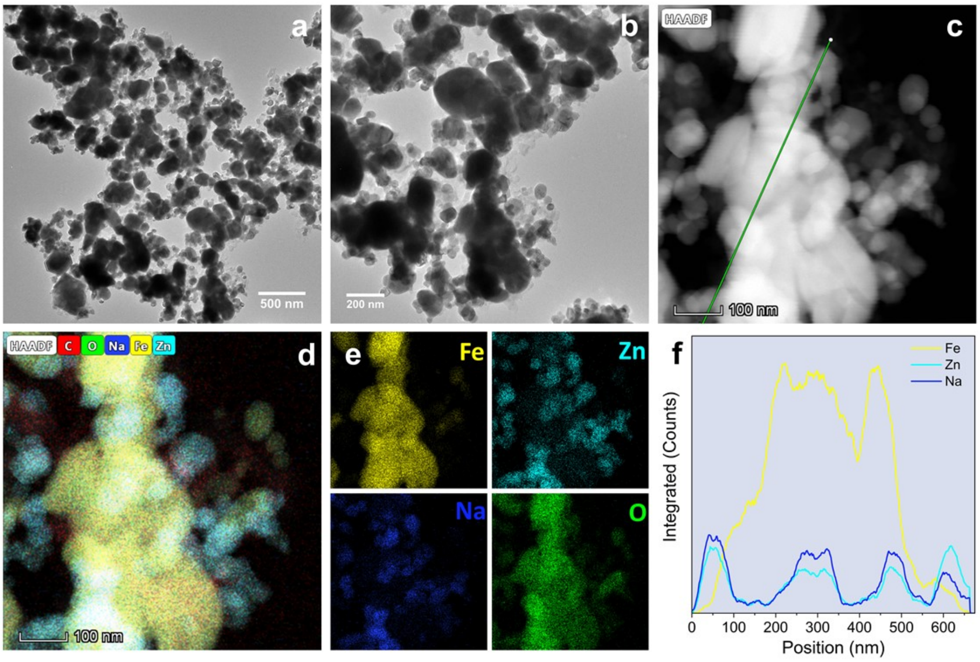

Fig. S7. The Morphology of the spent FeZnNa-I catalyst after reaction. (a-c) TEM images, (d-e) corresponding elemental mapping and (f) line scanning.

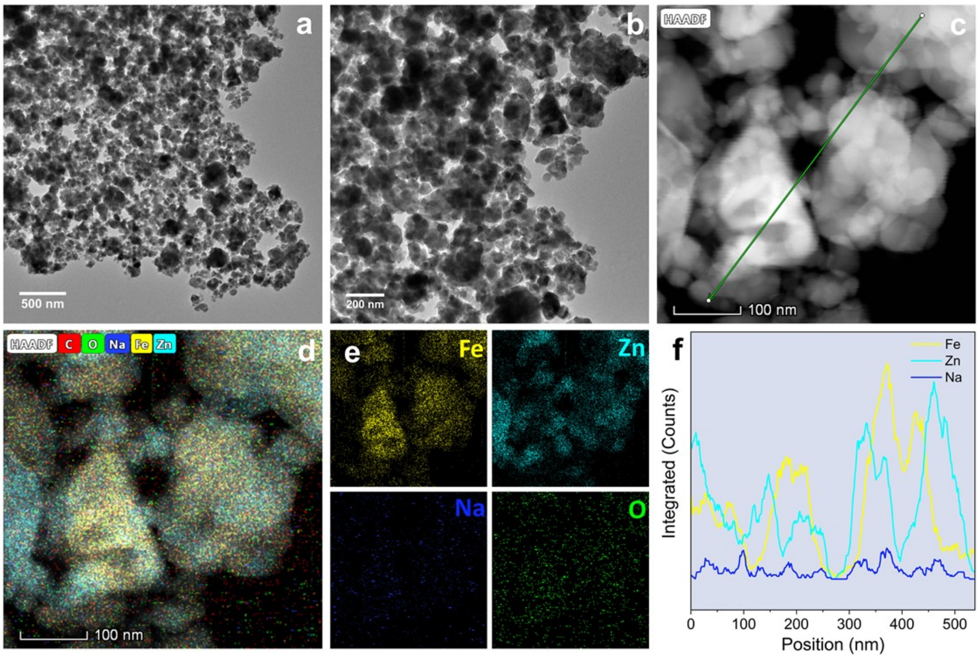

Fig. S8. The Morphology of the spent FeZnNa-C catalyst after reaction. (a-c) TEM images, (d-e) corresponding elemental mapping and (f) line scanning.

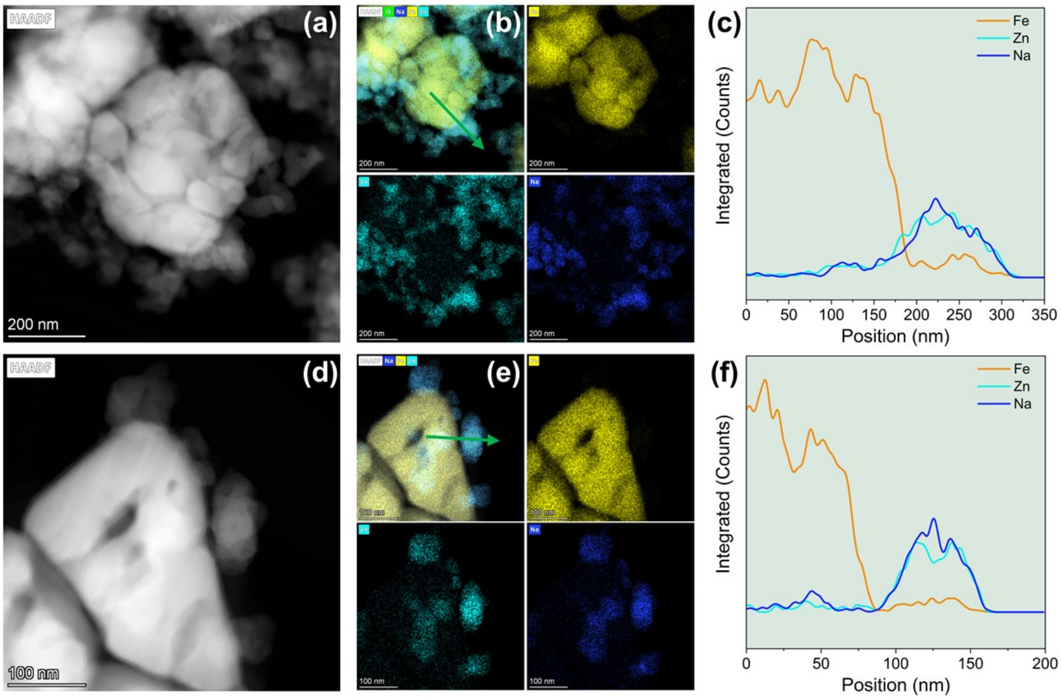

Fig. S9. The corresponding elemental mapping and line scanning profile of the spent FeZnNa-C catalyst.

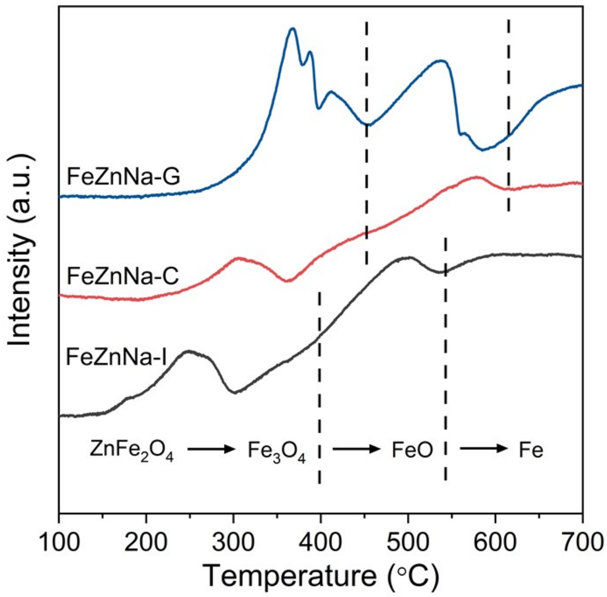

Fig. S10. H_2-TPR of the as-prepared catalysts.

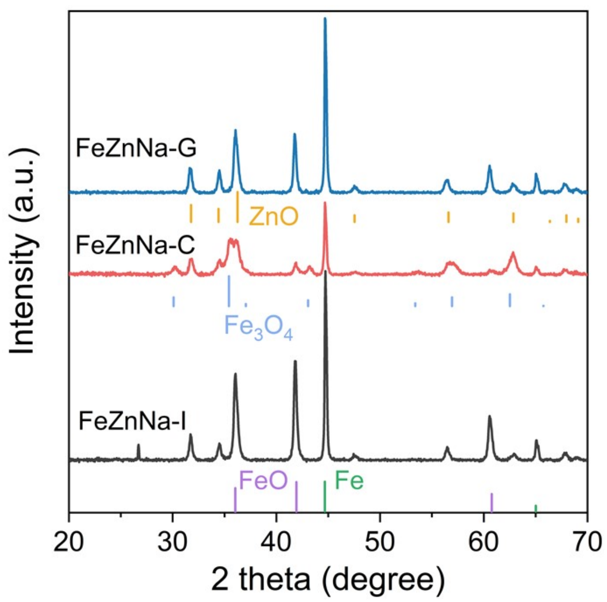

Fig. S11. XRD patterns of the catalysts after reduced.

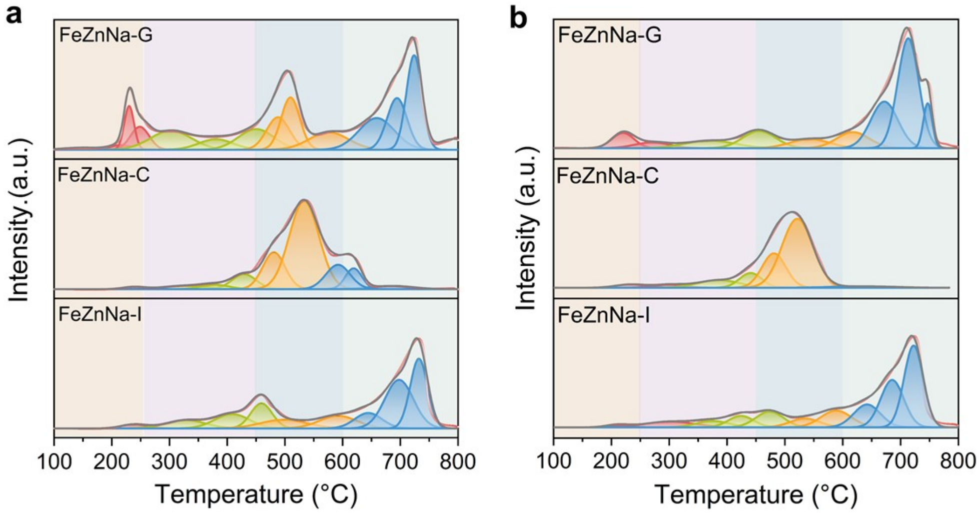

Fig. S12. (a) CO_2-TPD and (b) CO-TPD profiles of the spent catalysts.

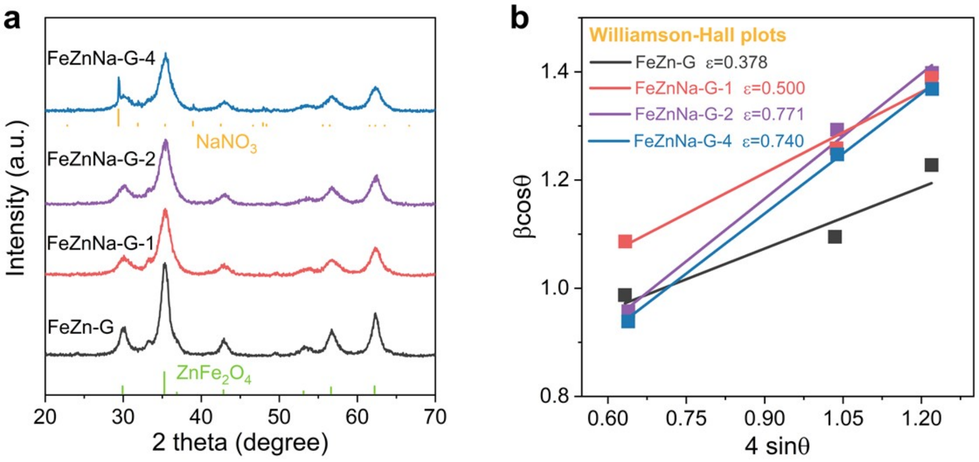

Fig. S13. (a) XRD patterns and (b) Williamson-Hall plots of the as-prepared FeZn catalysts with different Na content (1, 2, 4 represent 1%, 2% and 4%, respectively).

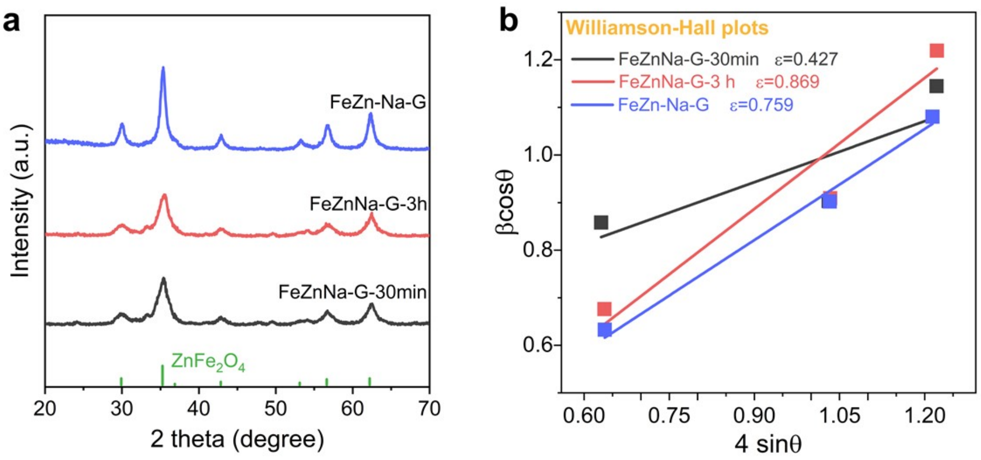

Fig. S14. (a) XRD patterns and (b)Williamson-Hall plots of the catalysts with different ball-milling time (30 min, 3 h represent ball-milling time) and preparation sequence (FeZn precursor was first synthesized, followed by ball milling Na into the precursor, which was named as FeZn-Na-G).

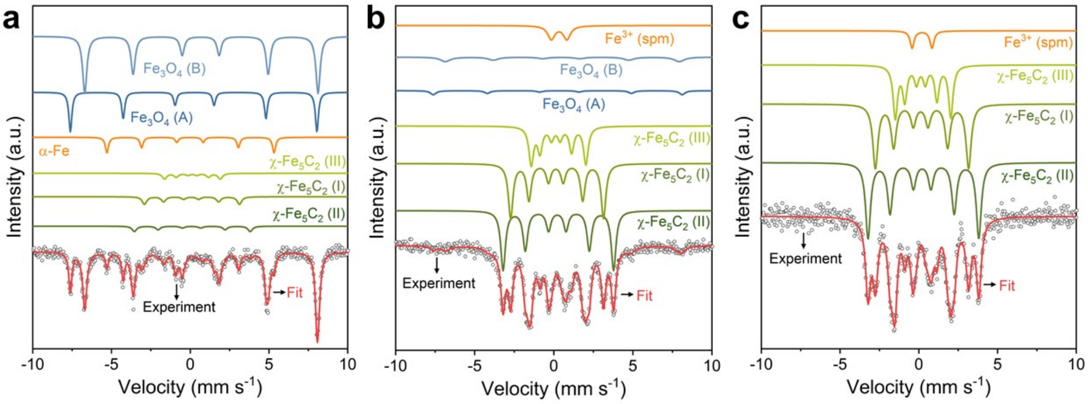

Fig. S15. Mössbauer spectra of the (a) FeZn-G, (b) FeZnNa-G-1, (c) FeZn-Na-G catalysts after reaction.

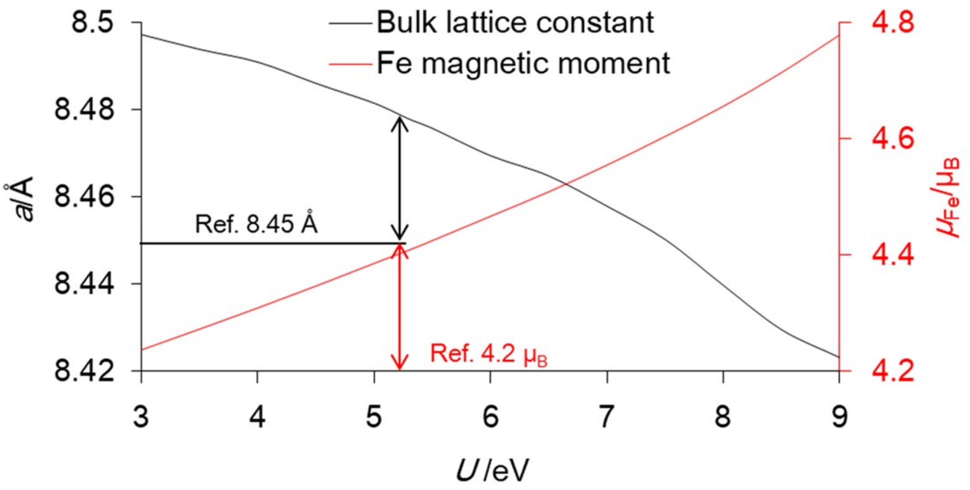

Fig. S16. The bulk lattice constant (a) of $ZnFe_2O_4$ and magnetic moment (μ) of Fe atom evaluated by DFT+U method.

Table S1. Elemental composition of the as-prepared catalysts determined by ICP-OES.

Catalysts	Fe contents (wt%)	Zn contents (wt%)	Na contents (wt%)
FeZnNa-I	57.0	8.3	1.3
FeZnNa-C	55.0	9.5	0.9
FeZnNa-G	56.7	9.6	2.0

Table S2. Catalytic performance for CO_2 hydrogenation at different reaction temperatures.

Catalysts	Temp.	CO_2 Conv. (%)	CO Select. (%)	Hydrocarbon Select. (%)				$STY_{C4+=}$ ($mg \cdot g_{cat}^{-1} \cdot h^{-1}$)
				CH_4	C_{2+}	$C_{2+=}$	$C_{4+=}$	
FeZnNa-I	280 °C	16.8	24.8	12.0	88.0	56.7	36.4	77.6
	300 °C	26.7	15.9	10.6	89.4	63.7	41.6	157.6
	320°C	32.5	12.4	11.9	88.1	65.7	43.8	210.4
FeZnNa-C	280 °C	14.3	26.0	20.7	79.3	33.6	18.6	33.2
	300 °C	24.9	16.1	19.7	80.3	47.8	29.6	104.4
	320°C	30.8	12.8	19.3	80.7	49.6	31.4	142.3
FeZnNa-G	280 °C	19.6	19.2	6.1	93.9	73.9	61.7	164.9
	300 °C	29.9	13.3	6.6	93.4	75.7	63.7	278.7
	320°C	38.2	10.2	7.1	92.9	76.3	64.0	370.5

Reduction conditions: H_2, 4000 $mL \cdot g^{-1} \cdot h^{-1}$, 340 °C, 2 h

Reaction conditions: H_2: CO_2: N_2=67.5:22.5:10, 2 MPa、12000 $mL \cdot g^{-1} \cdot h^{-1}$

Table S3. Reaction performance of catalysts for CO_2 hydrogenation.

Catalysts	CO_2 Conv. (%)	CO Select. (%)	Hydrocarbon Select. (%)				$STY_{C4+=}$ ($mg \cdot g_{cat}^{-1} \cdot h^{-1}$)
			CH_4	C_{2+}	$C_{2+=}$	$C_{4+=}$	
FeZnNa-I	42.8	8.8	11.5	88.5	62.1	47.6	104.5
FeZnNa-C	41.7	8.4	13.6	86.4	55.1	33.5	72.0
FeZnNa-G	47.9	6.9	8.3	91.7	74.8	63.5	159.3

Reduction conditions: H_2, 4000 $mL \cdot g^{-1} \cdot h^{-1}$, 340 °C, 2 h

Reaction conditions: H_2: CO_2: N_2=67.5:22.5:10、340 °C, 2 MPa、4000 $mL \cdot g^{-1} \cdot h^{-1}$

Table S4. Reaction performance of catalysts for CO_2 hydrogenation.

Catalysts	CO_2 Conv. (%)	CO Select. (%)	Hydrocarbon Select. (%)				$STY_{C4+=}$ ($mg \cdot g_{cat}^{-1} \cdot h^{-1}$)
			CH_4	C_{2+}	$C_{2+=}$	$C_{4+=}$	
FeZnNa-I	38.6	10.7	11.8	88.2	66.3	43.8	254.8
FeZnNa-C	35.4	10.4	19.3	80.7	54.1	34.3	183.6
FeZnNa-G	47.7	9.1	7.3	92.7	77.5	64.9	474.9

Reduction conditions: H_2, 4000 $mL \cdot g^{-1} \cdot h^{-1}$, 340 °C, 2 h
Reaction conditions: H_2: CO_2: N_2=67.5:22.5:10、340 °C, 2 MPa、12000 $mL \cdot g^{-1} \cdot h^{-1}$

Table S5. Comparison of the catalytic performance in CO_2 hydrogenation in literatures.

Catalysts	Temp. (℃)	Pressure (MPa)	GHSV (SL·g^{-1}·h^{-1})	CO_2 Conv. (%)	CO Select. (%)	Hydrocarbon Select. (%)			$STY_{C4+=}$ (mg·g_{cat}^{-1}·h^{-1})	Ref.
						CH_4	$C_{2+=}$	$C_{4+=}$		
K-Fe/ZrO_2	340	2.0	1.2	42.0	15.0	20.0	54.8	N.G.[g]	33.0[a]	(1)
FeZn-Na	320	3.0	4.0	37.5	11.5	15.0	45.9	N.G.[g]	85.7[a]	(2)
FeNaC-N_2	320	3.0	12.0	36.9	10.7	14.3	70.6	N.G.[g]	392.6[a]	(3)
FeMnNa	340	2.0	12.0	35.1	18.1	13.1	77.4	N.G.[g]	375.5[a]	(4)
NaSrFe	320	3.0	8.0	40.5	8.3	9.7	77.5	N.G.[g]	323.8[a]	(5)
Na/Fe_4N	320	1.5	10.0	29.8	31.4	32.9	60.5	N.G.[g]	173.9[a]	(6)
FeKMg	340	2.0	6.0	41.5	12.4	10.5	76.6	N.G.[g]	235.0[a]	(7)
Co1Fe2	320	2.0	8.0	40.9	9.5	11.0	72.5	N.G.[g]	301.9[a]	(8)
CoFe-33%CO	320	2.0	8.0	48	6.1	18.8	64.9	N.G.[g]	329.1[a]	(9)
FeK/SWNTs	320	2.0	9.0	52.7	9.6	13.5	62.3	47.5	286.4	(10)
NaFe/C	300	3.0	4.0	40.2	8.5	15.8	63.8	41.2	85.2	(11)
$CuFeO_2$	320	0.3	2.4	27.3	43.7	5.4	85.7	66.9	34.7	(12)
Fe/Co-Y_k	300	1.0	2.4	25.9	21.1	13.9	70.9	45.9	31.7	(13)
Fe/C-Bio	320	3.0	2.24	31.0	23.2	11.8	72.0	50.3	37.7	(14)
$FeAlO_x$-5	330	3.5	2.0	20.2	16.8	5.4	78.5	66.8	31.6	(15)
K-Fe15	300	0.5	2.7	45.0	12.5	18.3	72.3	30.0	44.9	(16)
FeZnNa-G	340	2.0	12.0	47.7	9.1	7.3	21.3	71.4	474.9	This work

[a]The STY of $C_{2+}^{=}$ olefins. [g]Not given.

Table S6. Textural properties of the as-prepared catalysts.

Catalysts	BET surface area ($m^2 \cdot g^{-1}$)	Pore volume ($cm^3 \cdot g^{-1}$)	Average pore size (nm)	d (nm)[a]
FeZnNa-I	83.8	0.17	5.8	12.5
FeZnNa-C	94.6	0.19	5.5	10.5
FeZnNa-G	48.4	0.10	5.7	8.2

[a]calculated via Scherrer equation

Table S7. Detailed Mössbauer parameters of the spent catalysts.

Catalyst	Assignment	IS (mm s^{-1})	QS (mm s^{-1})	Hhf (kOe)	Area (%)
FeZnNa-I	Fe_3O_4 (A)	0.25	-0.01	480	10.3
	Fe_3O_4 (B)	0.50	-0.01	440	20.5
	Fe_5C_2 (I)	0.10	0.07	184	20.6
	Fe_5C_2 (II)	0.22	0.09	219	17.5
	Fe_5C_2 (III)	0.18	0.10	110	12.5
	Fe^{3+}	0.31	0.70	—	9.8
	α-Fe	0.58	0.00	349	8.8
FeZnNa-C	Fe_3O_4 (A)	0.29	-0.02	485	16.9
	Fe_3O_4 (B)	0.63	-0.01	449	45.1
	Fe_5C_2 (I)	0.15	0.00	187	6.8
	Fe_5C_2 (II)	0.25	0.00	216	19.0
	Fe_5C_2 (III)	0.17	0.00	109	4.9
	Fe^{3+}	0.15	0.96	—	7.3
FeZnNa-G	Fe_5C_2 (I)	0.25	-0.08	185	36.3
	Fe_5C_2 (II)	0.25	0.10	217	36.0
	Fe_5C_2 (III)	0.13	-0.04	107	24.4
	Fe^{3+}	0.13	0.97	—	3.3
FeZn-G	Fe_3O_4 (A)	0.24	-0.06	486	24.4
	Fe_3O_4 (B)	0.68	0.04	460	47.6
	Fe_5C_2 (I)	0.08	0.05	187	9.8
	Fe_5C_2 (II)	0.10	0.04	218	4.8
	Fe_5C_2 (III)	0.13	0.00	110	6.9
	α-Fe	0.00	0.03	329	5.2
FeZnNa-G-1	Fe_3O_4 (A)	0.30	-0.10	489	2.7
	Fe_3O_4 (B)	0.50	0.08	459	5.8
	Fe_5C_2 (I)	0.17	0.07	182	30.9
	Fe_5C_2 (II)	0.25	0.06	217	33.6
	Fe_5C_2 (III)	0.21	0.17	107	21.4
	Fe^{3+}	0.33	0.98	—	5.6
FeZn-Na-G	Fe_5C_2 (I)	0.15	0.08	184	34.0
	Fe_5C_2 (II)	0.25	0.07	218	38.8
	Fe_5C_2 (III)	0.20	0.13	109	23.6
	Fe^{3+}	0.20	1.26	—	3.6

Table S8. Surface elemental composition of the as-prepared catalysts from XPS.

Catalysts	Fe	Zn	Na	Zn/Fe	Na/Fe
FeZnNa-I	24.65	4.67	2.17	0.19	0.09
FeZnNa-C	20.68	8.72	1.32	0.42	0.06
FeZnNa-G	17.39	7.09	7.16	0.41	0.40

Table S9. Surface elemental composition of the spent catalysts from XPS.

Catalysts	Fe	Zn	Na	Zn/Fe	Na/Fe
FeZnNa-I	4.00	7.13	3.99	1.78	0.99
FeZnNa-C	10.95	15.61	1.35	1.43	0.12
FeZnNa-G	3.58	10.35	10.45	2.89	2.92

Table S10. EXAFS fitting parameters at the Fe K-edge for various catalysts in reference to Fe foil and Fe_2O_3 (S_0^2=0.7).

Sample	Shell	N^a	R(Å)^b	σ^2(Å^2)^c	ΔE_0(eV)^d	R factor
Fe foil	Fe-Fe1	8.00	2.18	0.00543	-3.865	0.0080
	Fe-Fe2	6.00	3.09	0.00543	-3.865	
Fe_2O_3	Fe-O	2.00	1.82	0.04988	8.262	0.0186
	Fe-Fe	6.00	2.95	0.04988	8.262	
FeZnNa-I	Fe-O	7.50	1.98	0.00529	-4.552	0.0112
	Fe-Fe/Zn	5.00	2.96	0.00529	-4.552	
	Fe-Zn	5.50	3.47	0.00529	-4.552	
FeZnNa-C	Fe-O	8.00	1.99	0.00614	-4.273	0.0164
	Fe-Fe/Zn	6.20	2.97	0.00614	-4.273	
	Fe-Zn	7.00	3.48	0.00614	-4.273	
FeZnNa-G	Fe-O	8.00	1.97	0.00530	-4.192	0.0109
	Fe-Fe/Zn	6.50	2.94	0.00530	-4.192	
	Fe-Zn	5.20	3.44	0.00530	-4.192	

[a]CN, coordination number; [b]R, distance between absorber and backscatter atoms; [c]σ^2, Debye-Waller factor to account for both thermal and structural disorders; [d]ΔE_0, inner potential correction; R factor indicates the goodness of the fit; S_0^2 was set to 0.70. Note: The error range of CN and σ^2 is 20%, and the accuracy range of R is ± 0.03 Å. EXAFS data were fitted based on the $ZnFe_2O_4$ crystal structure.

Table S11. Reaction performance of catalysts for CO_2 hydrogenation.

Catalysts	CO_2 Conv. (%)	CO Select. (%)	Hydrocarbon Select. (%)				$STY_{C4+=}$ ($mg \cdot g_{cat}^{-1} \cdot h^{-1}$)
			CH_4	C_{2+}	$C_{2+=}$	$C_{4+=}$	
FeZn-G	31.9	12.7	24.0	76.0	12.9	9.8	46.1
FeZnNa-G-1	32.5	16.4	10.8	89.2	65.9	49	224.7
FeZnNa-G-3h	31.2	18.0	12.1	87.9	64.6	50.5	218.0
FeZn-Na-G	42.0	10.6	7.1	92.9	75.0	63.8	404.3

Reduction conditions: H_2, 4000 $mL \cdot g^{-1} \cdot h^{-1}$, 340 °C, 2 h

Reaction conditions: H_2: CO_2: N_2=67.5:22.5:10、340 °C, 2 MPa、12000 $mL \cdot g^{-1} \cdot h^{-1}$

SI References

1. J. Wang, Z. You, Q. Zhang, W. Deng, Y. Wang, Synthesis of lower olefins by hydrogenation of carbon dioxide over supported iron catalysts. *Catal. Today* **215**, 186–193 (2013).

2. H. Yang, *et al.*, Selective synthesis of olefins via CO_2 hydrogenation over transition-metal-doped iron-based catalysts. *Appl. Catal. B Environ.* **321**, 122050 (2023).

3. Y. Fu, *et al.*, EDTA chemical directly orient CO_2 hydrogenation towards olefins. *Chem. Eng. J.* **438**, 135597 (2022).

4. Y. Xu, *et al.*, Highly Selective Olefin Production from CO_2 Hydrogenation on Iron Catalysts: A Subtle Synergy between Manganese and Sodium Additives. *Angew. Chem. Int. Ed.* **132**, 21920–21928 (2020).

5. J. I. Orege, *et al.*, Highly stable Sr and Na co-decorated Fe catalyst for high-valued olefin synthesis from CO_2 hydrogenation. *Appl. Catal. B Environ.* **316**, 121640 (2022).

6. Z. Zhang, *et al.*, N Restructuring of Iron-Based Catalysts Boosting the Formation of C_{2+} Olefins from CO_2 Hydrogenation. *ACS Catal.* 8740–8752 (2025). https://doi.org/10.1021/acscatal.5c00267.

7. F. Qian, *et al.*, Stabilized Fe_7C_3 catalyst with K-Mg dual promotion for robust CO_2 hydrogenation to high-value olefins. *Nat Commun* **16**, 8044 (2025).

8. N. Liu, *et al.*, Elucidating the structural evolution of highly efficient Co-Fe bimetallic catalysts for the hydrogenation of CO_2 into olefins. *Appl. Catal. B Environ.* **328**, 122476 (2023).

9. N. Liu, *et al.*, Fine-tuning the active phases of CoFe alloy carbides for boosting olefin synthesis from CO_2 hydrogenation. *ACS Catal.* **15**, 179–192 (2025).

10. S. Wang, *et al.*, Iron–Potassium on Single-Walled Carbon Nanotubes as Efficient Catalyst for CO $_2$ Hydrogenation to Heavy Olefins. *ACS Catal.* **10**, 6389–6401 (2020).

11. C. C. Amoo, J. I. Orege, Q. Ge, J. Sun, Exploiting the latency of carbon as catalyst in CO_2 hydrogenation. *Chem. Eng. J.* **471**, 144606 (2023).

12. Z. Li, *et al.*, Ambient-pressure hydrogenation of CO_2 into long-chain olefins. *Nat Commun* **13**, 2396 (2022).

13. L. Guo, *et al.*, Selective formation of linear-alpha olefins (LAOs) by CO_2 hydrogenation over bimetallic Fe/Co-Y catalyst. *Catal. Commun.* **130**, 105759 (2019).

14. L. Guo, *et al.*, Directly converting carbon dioxide to linear α-olefins on bio-promoted catalysts. *Commun. Chem.* **1**, 11 (2018).

15. M. K. Khan, *et al.*, Selective Conversion of Carbon Dioxide into Liquid Hydrocarbons and Long-Chain α-Olefins over Fe-Amorphous AlO_x Bifunctional Catalysts. *ACS Catal.* **10**, 10325–10338 (2020).

16. C. G. Visconti, *et al.*, CO_2 hydrogenation to lower olefins on a high surface area K-promoted bulk Fe-catalyst. *Appl. Catal. B Environ.* **200**, 530–542 (2017).
